# Impact of the NO-Sensitive Guanylyl Cyclase 1 and 2 on Renal Blood Flow and Systemic Blood Pressure in Mice

**DOI:** 10.3390/ijms19040967

**Published:** 2018-03-23

**Authors:** Evanthia Mergia, Manuel Thieme, Henning Hoch, Georgios Daniil, Lydia Hering, Mina Yakoub, Christina Rebecca Scherbaum, Lars Christian Rump, Doris Koesling, Johannes Stegbauer

**Affiliations:** 1 Institute of Pharmacology and Toxicology, Medical Faculty, Ruhr-University Bochum, 44801 Bochum, Germany; mergia@outlook.de (E.M.); mergia@evanthia.de (G.D.); doris.koesling@ruhr-uni-bochum.de (D.K.); 2 Department of Nephrology, Medical Faculty, University Hospital Düsseldorf, Heinrich-Heine-University Düsseldorf, 40225 Düsseldorf, Germany; thieme.manuel@gmail.com (M.T.); henning.hoch@med.uni-duesseldorf.de (H.H.); lydia.hering@med.uni-duesseldorf.de(L.H.); mina.yakoub@med.uni-duesseldorf.de (M.Y.); ChristinaRebecca.scherbaum@med.uni-duesseldorf.de (C.R.S.); christian.rump@med.uni-duesseldorf.de (L.C.R.)

**Keywords:** renal blood flow, hypertension, NO-sensitive guanylyl cyclase, nitric oxide, cGMP, kidney, vasorelaxation, blood pressure, L-NAME, NO-GC

## Abstract

Nitric oxide (NO) modulates renal blood flow (RBF) and kidney function and is involved in blood pressure (BP) regulation predominantly via stimulation of the NO-sensitive guanylyl cyclase (NO-GC), existing in two isoforms, NO-GC1 and NO-GC2. Here, we used isoform-specific knockout (KO) mice and investigated their contribution to renal hemodynamics under normotensive and angiotensin II-induced hypertensive conditions. Stimulation of the NO-GCs by *S*-nitrosoglutathione (GSNO) reduced BP in normotensive and hypertensive wildtype (WT) and NO-GC2-KO mice more efficiently than in NO-GC1-KO. NO-induced increase of RBF in normotensive mice did not differ between the genotypes, but the respective increase under hypertensive conditions was impaired in NO-GC1-KO. Similarly, inhibition of endogenous NO increased BP and reduced RBF to a lesser extent in NO-GC1-KO than in NO-GC2-KO. These findings indicate NO-GC1 as a target of NO to normalize RBF in hypertension. As these effects were not completely abolished in NO-GC1-KO and renal cyclic guanosine monophosphate (cGMP) levels were decreased in both NO-GC1-KO and NO-GC2-KO, the results suggest an additional contribution of NO-GC2. Hence, NO-GC1 plays a predominant role in the regulation of BP and RBF, especially in hypertension. However, renal NO-GC2 appears to compensate the loss of NO-GC1, and is able to regulate renal hemodynamics under physiological conditions.

## 1. Introduction

To ensure renal function, renal blood flow (RBF) is kept constant during a wide range of systemic blood pressure levels due to adjustment of renal vascular resistance. An important regulator of vascular resistance is the nitric oxide (NO)/cyclic guanosine monophosphate (cGMP) signaling cascade that acts in smooth muscle cells and blunts the effects of vasoconstrictor factors [1,2,3]. A key enzyme in this pathway is the NO-sensitive guanylyl cyclase (NO-GC), which is stimulated by binding of NO to its prosthetic heme group and catalyzes, subsequently, the synthesis of the second messenger cGMP [4]. 

The NO-GC exists in two distinct isoforms, the NO-GC1 that corresponds to the α_1_β_1_ heterodimer and the NO-GC2 that refers to the α_2_β_1_ enzyme [5]. Since isoform-selective inhibitors do not exist, studies in knockout (KO) mice lacking the NO-GC1 or the NO-GC2 isoform are essential to elucidate their physiological roles [6]. Previous studies have suggested that the NO-GC1 is the predominant isoform in the systemic vasculature. The NO-GC2 plays a less important role as it accounts for a very low portion of total cGMP formation induced by NO (about 5% in the aorta) [6,7]. Yet, NO-GC2 is able to contribute to vascular relaxation induced by endothelial NO, as shown in NO-GC1-KO mice [6,7,8,9]. Additional deletion of NO-GC2 in smooth muscle cells resulted in hypertension by reducing vascular responsiveness to NO, which confirms the importance of NO-GC2 as a target for endothelial NO in the systemic vasculature [10]. 

In the kidney, NO modulates vascular resistance and has a profound impact on autoregulation of renal blood flow [11,12,13,14]. Deficiency of NO causes dysfunction of renal blood flow and promotes renal failure and hypertension [15]. In this context, a recent study has shown that activation of NO-GC by cinaciguat under NO-deficient conditions can normalize blood pressure and reduce renal vasoconstriction to improve renal blood flow autoregulation [16]. In addition, other studies indicate NO-GC as an emerging target for the treatment of renal disorders [17]. 

Similarly, inhibition of the cGMP-degrading phosphodiesterase 5 (PDE5) enhances cGMP levels, which results in an increase in RBF, improved renal vascular function, and lower blood pressure in experimental models of hypertension [7,14,17,18].

In order to get more insights into how the NO/cGMP pathway regulates renal hemodynamics, we investigated the relative contribution of the NO-GC1 and NO-GC2 isoforms on renal blood flow, renal vascular function, and blood pressure regulation by using NO-GC1-KO and NO-GC2-KO mice.

## 2. Results

### 2.1. Renal Blood Flow under Basal Conditions

**Figure 1:**
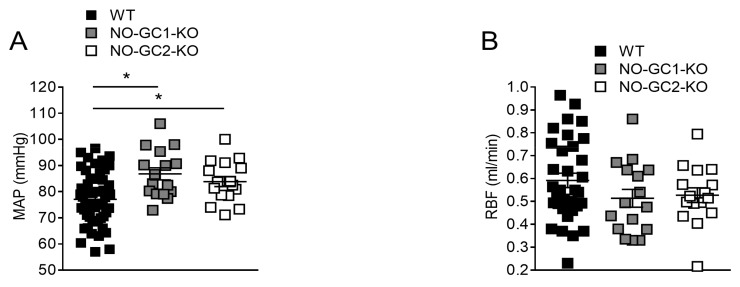
Increased blood pressure but normal renal blood flow in anesthetized NO-sensitive guanylyl cyclase1 knockout (NO-GC1-KO) and NO-GC2-KO mice. (**A**) Blood pressure was significantly increased in NO-GC1-KO (*n* = 16) and NO-GC2-KO (*n* = 17) compared to WT (*n* = 54) mice; (**B**) Renal blood flow was not different in unconscious WT (*n* = 33), NO-GC1-KO (*n* = 16) and NO-GC2-KO (*n* = 16) mice. Data represent means ± standard error of the mean (SEM); * *p* < 0.05 vs. wildtype (WT). One-way standard error of the mean (ANOVA) followed by Bonferroni’s multiple comparison post hoc test.

In order to investigate the impact of NO-GC isoforms on renal hemodynamics, we measured renal blood flow in unconscious WT, NO-GC1-KO, and NO-GC2-KO mice. Interestingly, under anesthesia, invasively measured mean arterial blood pressure (MAP) was significantly increased in both NO-GC1-KO (86 ± 2 mmHg) and NO-GC2-KO (84 ± 2 mmHg) compared to WT mice (77 ± 1 mmHg) indicating that both isoforms contribute to systemic vascular resistance (Figure 1A). Nevertheless, renal blood flow was unaltered in NO-GC1-KO (0.51 ± 0.04 mL/min) and NO-GC2-KO (0.53 ± 0.03 mL/min) compared to WT (0.59 ± 0.03 mL/min) mice (Figure 1B). These observations suggest preserved renal blood flow autoregulation in NO-GC1-KO and NO-GC2-KO mice under basal conditions.

### 2.2. Effects of NO-sensitive guanylyl cyclase (NO-GC)1 and NO-GC2 on Renal Blood Flow in Response to NO Stimulation

**Figure 2:**
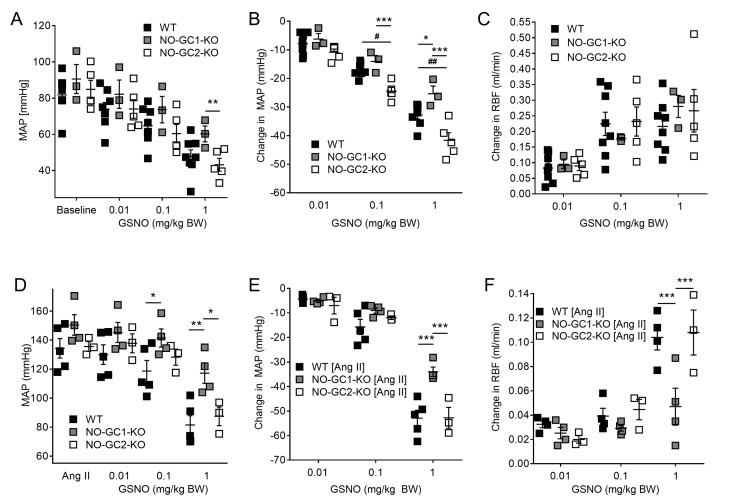
Predominant role of NO-GC1 in GSNO-induced blood pressure reduction and renal blood flow increase in anesthetized mice. (**A**,**B**) In normotensive mice, GSNO induced blood pressure reduction, expressed as absolute blood pressure values and blood pressure reduction, was attenuated in NO-GC1-KO (*n* = 3) compared to WT (*n* = 8) and NO-GC2-KO (*n* = 5) mice; (**C**) In normotensive mice, GSNO-induced increase in renal blood flow did not differ between the three genotypes (*n* = 3–8); (**D**–**F**) In Ang II-infused mice (200 ng/kg/min), GSNO-induced blood pressure reduction, expressed as absolute blood pressure values and blood pressure reduction, and renal blood flow increase were significantly attenuated in NO-GC1-KO (*n* = 3) compared to WT (*n* = 5) and NO-GC2-KO (*n* = 4) mice. * *p* < 0.05, ** *p* < 0.01, *** *p* < 0.001 vs. NO-GC1-KO; # *p* < 0.01, ## *p* < 0.001 vs. NO-GC2-KO. Two-way ANOVA followed by Bonferroni’s multiple comparison post hoc test.

To further investigate the role of NO-GC1 and NO-GC2 in renal hemodynamics, we applied S-nitrosoglutathione (GSNO) (0.01–0.1–1.0 mg/kg body weight (BW)) intravenously, and measured changes in blood pressure and renal blood flow under baseline conditions. GSNO induced a concentration dependent decrease in blood pressure in WT, NO-GC1-KO, and NO-GC2-KO mice (Figure 2A,B). The highest GSNO concentration decreased blood pressure to hypotensive values in all three groups (WT: 49 ± 4; NO-GC-1-KO: 58 ± 4; NO-GC2-KO 43 ± 4 mmHg). GSNO-induced stimulation of the NO-GC2, the minor isoform in the vasculature, resulted in an attenuated blood pressure reduction in NO-GC1-KO mice compared to WT and NO-GC2-KO mice (Figure 2A,B). Interestingly, GSNO-induced increase of renal blood flow did not differ between the three groups and did not further increase at the highest GSNO concentration, most likely due to hypotension (Figure 2C). To exclude any pathophysiological effects of hypotension and to unmask the impact of the NO-GC isoforms on renal blood flow, we tested the effect of GSNO in mice infused with angiotensin II. During angiotensin II infusion, blood pressures increased, and did not differ between the three groups (WT: 134 ± 7; NO-GC1-KO: 150 ± 7; NO-GC2-KO: 136 ± 3 mmHg; Figure 2D). Similar to the effects described above, GSNO-induced (1 mg/kg BW) stimulation of both NO-GC isoforms in WT or NO-GC1 in NO-GC2-KO mice resulted in a significantly greater blood pressure reduction compared to GSNO-induced NO-GC2 stimulation in NO-GC1-KO mice (WT: 53 ± 3; NO-GC1-KO: 34 ± 2; NO-GC2-KO: 53 ± 4 mmHg; Figure 2E). In accordance with attenuated blood pressure reduction in NO-GC1-KO mice, GSNO-induced increase of renal blood flow was significantly smaller in mice lacking the NO-GC1 isoform, suggesting a major role of the NO-GC1 isoform in renal blood flow regulation (Figure 2F).

### 2.3. Inhibition of NO Production by L-NAME Affects Renal Blood Flow in NO-GC1-KO and NO-GC2-KO Mice

**Figure 3:**
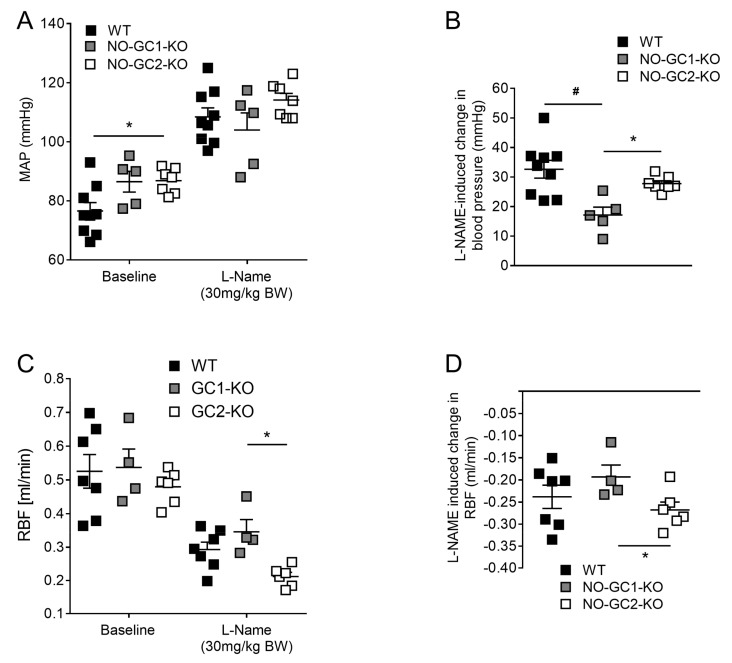
Inhibition of NO production by L-NAME affects blood pressure and renal blood flow in NO-GC1-KO and NO-GC2-KO mice. (**A**) In anesthetized mice, administration of L-NAME increased blood pressure to the same level; (**B**) L-NAME-induced blood pressure increase was significantly attenuated in NO-GC1-KO (*n* = 5) and NO-GC2-KO (*n* = 7) compared to WT (*n* = 9) mice; (**C**) Renal blood flow was significantly decreased by L-NAME in WT (*n* = 7), NO-GC1 (*n* = 4) and NO-GC2-KO (*n* = 6) mice; (**D**) L-NAME-induced reduction in renal blood flow was significantly reduced in NO-GC1-KO compared to NO-GC2-KO mice (*n* = 4–7). * *p* < 0.05; # *p* < 0.01 vs. NO-GC2-KO. One-way ANOVA or two-way ANOVA followed by Bonferroni’s multiple comparison post hoc test.

Infusions of the nitric oxide synthase inhibitor N(G)-Nitro-l-arginine methyl ester (L-NAME) (30 mg/kg BW, iv) were administrated to inhibit endogenous NO production. L-NAME increased MAP to a level that did not significantly differ between the three genotypes (WT: 108 ± 3; NO-GC1-KO: 104 ± 6; NO-GC2-KO: 114 ± 3 mmHg; Figure 3A). Yet, the increase of MAP induced by L-NAME was significant attenuated in NO-GC1-KO mice compared to WT and NO-GC2-KO mice (Figure 3B). Concomitant to the increase in blood pressure, renal blood flow decreased in the presence of L-NAME in all three genotypes (Figure 3C,D). These findings indicate that both NO-GC1 and NO-GC2 mediate the effects of endogenous NO on renal blood flow.

### 2.4. Contribution of NO-GC1 and NO-GC2 to Renal Vascular Relaxation

**Figure 4:**
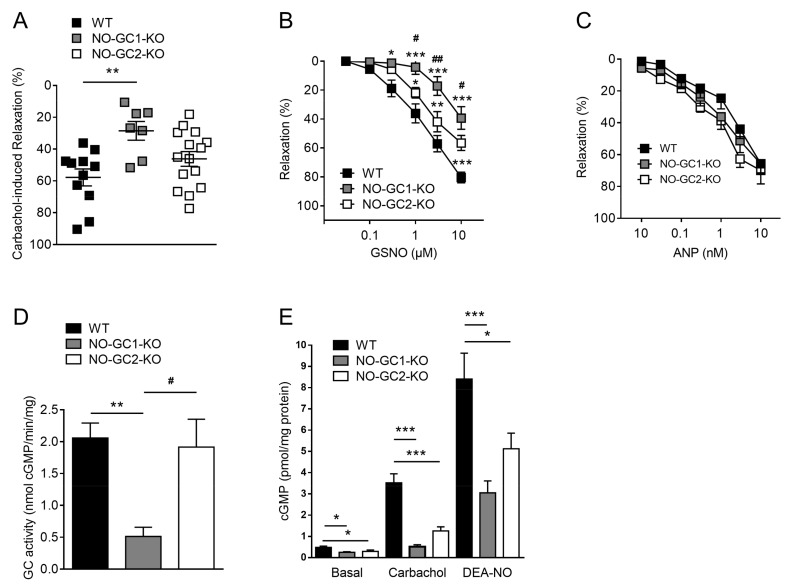
Impaired NO-mediated renal vasorelaxation in NO-GC1-KO and NO-GC2-KO mice. (**A**) Endothelial-dependent renal vasorelaxation by carbachol (30 µM) was significantly reduced in isolated perfused kidneys of NO-GC1-KO compared to WT mice; (**B**) Smooth muscle cell-dependent vasorelaxation induced by the NO donor GSNO was significantly attenuated in isolated perfused kidneys of NO-GC1-KO (*n* = 8) and NO-GC2-KO (*n* = 13) compared to WT (*n* = 11) mice; (**C**) Atrial natriuretic peptide (ANP)-induced renal vasorelaxation did not differ between WT (*n* = 6), NO-GC1-KO (*n* = 10) and NO-GC2-KO (*n* = 5) mice; (**D**) Renal NO-GC activity was significantly decreased in NO-GC1-KO (*n* = 6) compared to WT (*n* = 19) and NO-GC2-KO (*n* = 7) mice; (**E**) Renal cGMP levels at baseline, in response to carbachol (30 µM) or DEA-NO (100 µM) were significantly decreased in NO-GC1-KO (10 slices of *n* = 3) and NO-GC2-KO (11 slices of *n* = 3) compared to WT (10 slices of *n* = 3) mice. * *p* < 0.05, ** *p* < 0.01, *** *p* < 0.001 vs. WT; # *p* < 0.05, ## *p* < 0.01 vs. NO-GC1-KO-mice. Two-way ANOVA followed by Bonferroni’s multiple comparison post hoc test or one-way ANOVA followed by Bonferroni’s multiple comparison post hoc test.

To determine the relative contribution of NO-GC1 and NO-GC2 to renal vascular resistance independent from systemic blood pressure changes, we measured vasorelaxation ex vivo in isolated perfused kidneys. To characterize the contribution of the NO-GC isoforms in the signaling of endogenous NO, carbachol was applied at a maximally effective concentration (30 µM). Vasorelaxation induced by carbachol was significantly reduced in kidneys of NO-GC1-KO mice compared to WT and NO-GC2-KO mice (Figure 4A).

Similarly, renal vasorelaxation in response to exogenous NO, GSNO, was significantly attenuated in NO-GC1-KO compared to WT and NO-GC2-KO mice. In addition, GSNO induced renal vasorelaxation was also reduced in NO-GC2-KO compared WT mice (Figure 4B). Our findings suggest that NO-GC1 is the major target of NO in renal vasculature, but also demonstrate a substantial role of NO-GC2 in renal vasorelaxation. To determine if cGMP produced by the membrane-bound guanylyl cyclase GC-A compensates, in part, to the reduced renal vasorelaxation towards NO, ANP-induced renal relaxation was examined in all three genotypes. As shown in Figure 4C, renal vasorelaxation in response to ANP did not differ between WT, NO-GC1-KO, and NO-GC2-KO mice. This finding indicates that no quantifiable compensation takes place on the level of GC-A or of downstream cGMP effectors.

### 2.5. NO-Stimulated cGMP Formation in Kidney Homogenates and Cortical Slices

To assess the amount of the NO-GCs in the kidney, we measured NO-stimulated GC activity in whole kidney homogenates of WT and KO mice deficient of NO-GC1 or NO-GC2. In the NO-GC1-KO mice, the residual NO-GC activity is due to NO-GC2 content and amounted to 20% of the NO-GC activity in WT (Figure 4D). Yet, the portion of NO-GC2 was too low to detect a reduction of the NO-GC content in the NO-GC2-KO kidneys. 

We further studied the contribution of the NO-GCs to cGMP formation by measuring cGMP formation in renal cortical slices (Figure 4E). In untreated conditions, cGMP formation was found to be reduced in both NO-GC-KO lines by about 50% compared to WT (NO-GC1-KO: 0.26 ± 0.03; NO-GC2-KO: 0.30 ± 0.06; WT: 0.49 ± 0.06 pmol cGMP/mg protein). In response to carbachol (30 µM, 3 min), which induces eNOS-mediated NO formation, cGMP levels increased, but remained significantly reduced in the renal cortical slices of NO-GC1-KO and NO-GC2 KO mice compared to WT mice (NO-GC1-KO: 0.5 ± 0.09; NO-GC2-KO: 1.7 ± 0.46; WT: 3.5 ± 0.43 pmol cGMP/mg protein). Accordingly, administration of exogenous NO by the NO donor DEA-NO (100 µM, 3 min) induced an attenuated cGMP increase in renal slices of NO-GC1-KO and NO-GC2-KO mice compared to WT (NO-GC1 KO: 3 ± 0.56; NO-GC2 KO: 5.1 ± 0.74; WT: 8.4 ± 1.22 pmol cGMP/mg protein) mice. In summary, these experiments demonstrate the contribution of both NO-GC isoforms to cGMP formation in response to NO.

### 2.6. Blood Pressure Monitoring in Freely Moving NO-GC1-KO and NO-GC2-KO Mice

**Figure 5:**
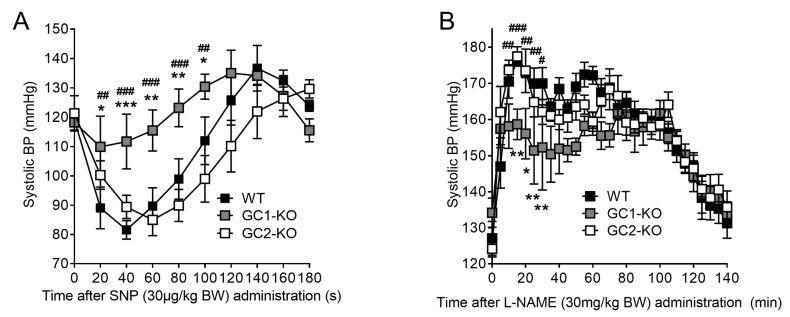

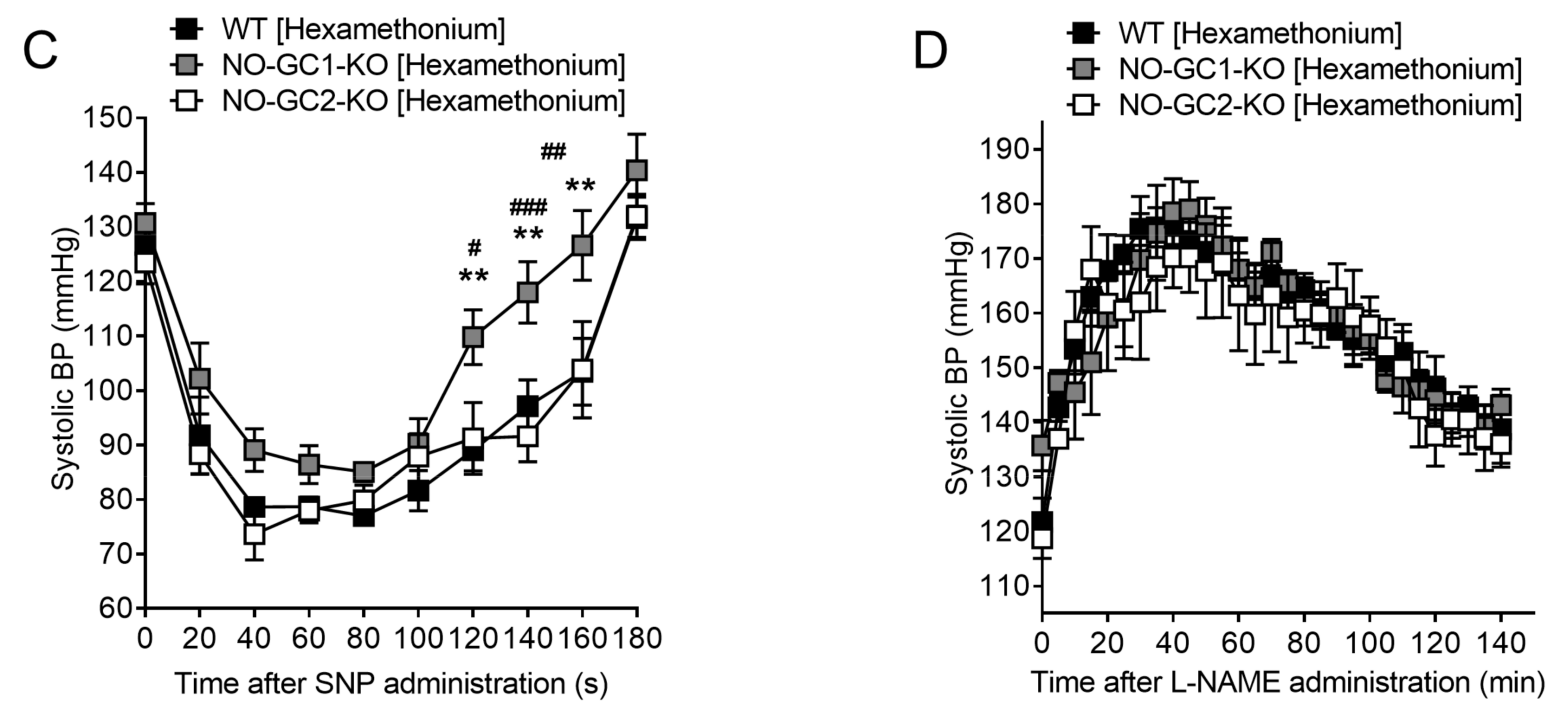
Predominant role of NO-GC1 in NO-mediated blood pressure changes in conscious mice. (**A**) Blood pressure reduction induced by the NO donor sodium nitroprusside (SNP) (30 µg/kg BW) was significantly diminished in conscious NO-GC1-KO (*n* = 4) mice compared to WT (*n* = 4) and NO-GC2-KO (*n* = 4) mice measured by radio telemetry; (**B**) Attenuated blood pressure increase by L-NAME (30 mg/kg BW) in NO-GC1-KO mice compared to WT and NO-GC2-KO mice; (**C**,**D**) Co-administration of hexamethonium (30 mg/kg BW) enhanced blood pressure reduction by GSNO and blood pressure increase by L-NAME in NO-GC1-KO (*n* = 6) compared to WT (*n* = 6) and NO-GC2-KO (*n* = 6) mice. * *p* < 0.05, ** *p* < 0.01, *** *p* < 0.001 vs. WT; # *p* < 0.05, ## *p* < 0.01, ### *p* < 0.001 vs. NO-GC2-KO-mice. Two-way ANOVA followed by Bonferroni’s multiple comparison post hoc test.

Finally, we studied the role of NO-GC1 and NO-GC2 in blood pressure regulation via radio telemetry in WT and KO mice. Baseline blood pressure did not differ between freely moving WT, NO-GC1-KO, and NO-GC2-KO mice (127 ± 3 vs. 132 ± 4 vs. 123 ± 3 mmHg, *n* = 6). Yet, comparable to the results seen in anesthetized mice, blood pressure reduction induced by the NO donor sodium nitroprusside (SNP) was significantly diminished in conscious NO-GC1-KO mice compared to WT and NO-GC2-KO (Figure 5A). This finding confirms NO-GC1 as the major NO-GC isoform mediating the vasodilatory effect of NO. In contrast to SNP, which acts by stimulating both NO-GCs, the general NOS inhibitor, L-NAME, acts by offsetting NO-GC activity. In contrast to the results with SNP, NOS inhibition by L-NAME increased blood pressure in all three genotypes (Figure 5B). Interestingly, the maximal blood pressure effect induced by L-NAME treatment was significantly smaller in NO-GC1-KO than in WT and NO-GC2-KO mice. These differences were not observed when the ganglionic blocker hexamethonium (30 mg/kg BW) has been co-administrated. This suggests that NO-GC1 and NO-GC2 regulate blood pressure not only through vascular but most probably also through central nervous effects (Figure 5C,D).

## 3. Discussion

In the present study, we examined the impact of NO-GC1 and NO-GC2 on renal vascular function and blood pressure regulation. NO-GC is the main target mediating the modulatory effects of nitric oxide in the kidney. Recent studies demonstrated that the NO-GC plays an important role in the pathogenesis of chronic kidney disease through both hemodynamic and direct pro-fibrotic effects [15,17,19,20,21]. However, the two distinct NO-GC isoforms have indistinguishable enzymatic properties and isoform-specific inhibitors are lacking. Therefore, not much attention has been paid to the role of NO-GCs in renal hemodynamics [5]. In contrast, many pharmacological or genetic approaches have been used to study the different NO synthase isoforms, eNOS, nNOS, or iNOS in kidney [13,15,22,23]. By analyzing KO mice deficient for each of the NO-GC isoforms, we were able to demonstrate a predominant role of the NO-GC1 isoform in regulating renal hemodynamics. In addition, we could also show a relative contribution of NO-GC2 to the regulation of blood pressure and RBF under normotensive condition. 

In agreement with our previous study, NO-GC1 was found to be the major NO-GC isoform expressed in the kidney, accounting for about 80% of the NO-stimulated GC activity [7]. In relation to NO-GC1, the amount of NO-GC2 appears negligible. Accordantly, NO-GC activity measured in kidneys of NO-GC2-KO mice did not differ from WT. Nevertheless, here we demonstrate that cGMP levels in renal cortical slices were reduced in both KO lines under baseline and NO-stimulated conditions. These results suggest a participation of both isoforms in the NO/cGMP signaling in the kidney. In line with this observation, both NO-GC isoforms were found to participate in NO-dependent vasorelaxation in isolated perfused kidneys. Yet, the NO-GC2 was not sufficient to fulfil a WT-like effect, most likely due to a lower cGMP formation. Taken together, our data demonstrate the participation of both NO-GC isoforms in mediating the renal vasodilator action of endothelial NO.

Previously, it has been reported that an enhanced vasorelaxation mediated by the membrane-bound GC-A in response to ANP was able to partly compensate the reduced vasorelaxation in aortas of NO-GC1-KO mice [6]. In the present study, ANP-induced renal vasorelaxation did not differ between WT and KO mice, and we excluded any differences on GC-A levels or downstream to cGMP formation in kidneys of NO-GC1-KO or NO-GC2-KO mice. 

Despite the influence of both NO-GCs on renal vasorelaxation, baseline renal blood flow was neither altered in NO-GC1-KO nor in NO-GC2-KO mice. By contrast, blood pressure was increased in both KO lines under anesthesia. This observation indicates a sufficient autoregulatory response in the absence of either NO-GC1 or NO-GC2, and is in accordance with previous studies showing normal baseline blood flow despite hypertension in eNOS KO mice [13,22,24].

To directly address the contribution of the NO-GC isoforms to blood pressure and renal blood flow, we stimulated the NO-GCs by applying exogenous NO (GSNO). Stimulation of the NO-GCs by GSNO induced a prominent blood pressure reduction in WT and NO-GC2-KO mice, which was attenuated in NO-GC1-KO. The lacking effect of NO to efficiently lower blood pressure in NO-GC1-KO mice is consistent with a lower content of NO-GC2 in the systemic vasculature, and consequently, lower increase of cGMP. The prominent vasodilating effect of NO induced an increase of renal blood flow which did not differ between the three genotypes. This finding suggests that an increase in renal blood flow under normotensive or hypotensive conditions is most likely mediated by a very low amount of cGMP, which can be supplied interchangeably either by NO-GC1 or by NO-GC2. Similarly, application of the NO-GC activator cinaciguat (BAY 58-2667), which induces a rather low cGMP increase, decreases BP without affecting renal blood flow in healthy rats [16,25].

Under hypertensive conditions induced by Ang II infusion, it becomes more obvious that the cGMP increase by the NO-GC2 is not capable to lower BP to the same extent as the cGMP increase induced by the NO-GC1. Accordingly, NO-GC2 failed to increase renal blood flow, as shown in hypertensive NO-GC1-KO mice. In comparison, NO led to a similar increase of renal blood flow in hypertensive WT and NO-GC2-KO mice. Thus, in hypertensive conditions, cGMP-formed by the NO-GC1 is needed to regulate BP and renal blood flow. 

The contribution of the NO-GC isoforms mediating the effects of endothelial NO in mice was examined by inhibition of endogenous NO production by L-NAME. As demonstrated in many studies, L-NAME increases blood pressure, and yet lowers renal blood flow by shifting the balance of the regulatory mechanisms toward a stronger vasoconstriction. In the eNOS-KO mice, L-NAME did not alter blood pressure or baseline blood flow, indicating that the presence of eNOS is mandatory for the L-NAME effects [13,22,24]. Compared to this observation, L-NAME alters blood pressure and renal blood flow in NO-GC1-KO and NO-GC2-KO mice by inhibiting NO stimulation of the residual isoform (NO-GC2 or NO-GC1, respectively). Thus, the effects obtained with L-NAME in our KO mice reveal the contribution of the residual NO-GC isoform in the regulation of blood pressure and renal blood flow. Inhibition of NO production increased blood pressure and reduced renal blood flow to a lower extent in NO-GC1-KO mice compared to WT and NO-GC2-KO mice.

Taken together, our study shows that NO-GC1 is the predominant target of endothelial NO in the systemic and renal vasculature. Nevertheless, we could also uncover a substantial contribution of the NO-GC2 to mediating NO effects on blood pressure and renal blood flow in vivo. Thus, the present study highlights the NO-GC as a master regulator of renal hemodynamics and blood pressure and therefore as an interesting new drug target in hypertension and kidney disease.

## 4. Material and Methods

### 4.1. Animal Models

Experiments were performed with male wild-type C57Bl/6J, NO-GC1-KO, and NO-GC2-KO mice (10–14 weeks old). The KO mice were generated and genotyped as described previously [6]. Mice experiments were approved by the responsible authority (Landesamt fuer Natur-, Umwelt- und Verbraucherschutz Nordrhein-Westfalen; reference: 87-51.04.2010.A039 (01.05.2010) and 8.87-50.10.34.08.216 (01.11.2008) and performed according to the guidelines from Directive 2010/63/EU of the European Parliament on the protection of animals used for scientific purposes.

### 4.2. Acute Blood Pressure Response and Changes in Renal Blood Flow

Acute pressor responses and renal blood flow to L-NAME and GSNO were measured in anesthetized WT, NO-GC1-KO, or NO-GC2-KO mice, as described previously [26]. In brief, mice were anesthetized intraperitoneally (ip) with ketamine (100 mg·kg^−1^) and xylazine (5 mg·kg^−1^), and decapitated at the end of the experiment. Mean arterial blood pressure was monitored continuously through a catheter placed in the right common carotid artery. Basal fluids, GSNO and L-NAME were administered via a catheter placed in the right jugular vein. Ang II (200 ng/kg/min) was applied continuously throughout the experiment via a second catheter placed in the left jugular vein. For measuring RBF, a small incision was made on the left flank and the left renal artery was dissected. An ultrasonic flowmeter interfaced with a 5 mm V-shaped probe was then placed around the left renal artery (MA0.5PSB and TS420 Flowmeter, Transonic Systems Inc., Ithaca, NY, USA). After a stabilization period of 30 min, the effects of GSNO and L-NAME were tested under normotensive conditions. Therefore, GSNO was administrated in increasing doses (0.01, 0.1, 1.0 mg/kg BW) at 5 min intervals. L-NAME was administrated at a dose of 0.03 mg/kg BW. Intra-arterial pressure and renal blood flow were monitored continuously using the PowerLab data acquisition system and LabChart software (ADInstruments, Colorado Springs, CO, USA). Changes in BP or RBF were recorded as the delta of BP- or RBF-increase or decrease in relation to their baseline values determined before the application of either GSNO or L-NAME.

### 4.3. Isolated Perfused Kidneys

Kidneys of WT, NO-GC1-KO, or NO-GC2-KO were isolated and perfused with Krebs–Henseleit buffer as described previously [27]. Changes in perfusion pressure reflected changes in vascular resistance of renal vessels. Immediately after preparation, a bolus of 60 mM KCl was injected to test the viability of the preparation followed by a stabilization period of 30 min. To assess renal vasodilation, kidneys were preconstricted with norepinephrine (1 µM; Sigma Aldrich, Taufkirchen, Germany). Concentration–response curves induced by GSNO (Alexis Corp., Enzo Life Sciences AG, Lausen, Germany) and ANP (Sigma Aldrich) were recorded in presence of diclofenac (3 µM; Sigma Aldrich) and L-NAME (300 µM; Sigma Aldrich). Vasodilation induced by carbachol (30 µM) was tested in the presence of diclofenac (3 µM). Renal relaxation is expressed as a percentage pressor response of the preconstricted kidney, which was set as 100%.

### 4.4. Blood Pressure Measurements in Freely Moving WT, NO-GC1-KO, and NO-GC2-KO Mice

In order to measure the blood pressure effects of the NO donor, SNP, or the NO synthase inhibitor, L-NAME, in conscious unrestrained mice, radio telemetry catheters (Data Sciences International, PA-C10, s’Hertogenbosch, The Netherlands) were implanted as described previously [28]. For catheter implantation, mice were anesthetized intraperitoneally with ketamine and xylazine (100 and 10 mg/kg, respectively), and the left common carotid artery was dissected. The artery was cannulated, and the catheter was advanced to the point where the small notch on the tubing resided at the vessel opening. Finally, the catheter was fixed, and the transmitter placed subcutaneously. After radio telemetry catheter implantation, mice were allowed to recover for seven days to re-establish normal circadian rhythms. For habituation, before the experiment, mice were trained daily (ip. injection of an equal amount of saline) for three consecutive days. Additionally, ip. injections were always performed between 8.00 and 10.00 a.m. Blood pressure levels were recorded continuously with measuring every 20 min for 10 s intervals. Thirty minutes before and 2 h after administration of SNP or L-NAME, blood pressure levels were recorded every 20 s for 10 s intervals. SNP (30 µg/kg BW), L-NAME (50 mg/kg BW), or a combination of hexamethonium (30 mg/kg BW) and SNP (30 µg/kg BW) or L-NAME (30 mg/kg BW) were administrated ip. once per mouse every 24 h for three days. The mean of three measurements per mouse was analyzed.

### 4.5. Measurement of cGMP Content and NO-Stimulated GC-Activity in Renal Cortical Slices

NO-stimulated GC activity was determined in kidney homogenates in the presence of 100 µM DEA-NO (2-(*N*,*N*-diethylamino)-diazenolate-2-oxide, Alexis Corp.), as described previously [6]. For measuring cGMP changes ex vivo, cortical slices (250 µm) were cut with a vibratome (NVSLM1 from WPI) and equilibrated for 10 min in tempered (37 °C), oxygenated (with 95% O_2_, 5% CO_2_) Krebs–Henseleit buffer as described previously [7]. To increase cGMP, cortical slides were incubated with carbachol (30 µM) or DEA-NO (100 µM) for 3 min. cGMP levels of equilibrated untreated slices were taken as baseline measurements. After incubation, slices were snap frozen in liquid nitrogen, homogenized in 70% ice-cold ethanol using a glass/glass homogenizer, and then centrifuged (20,000× *g*, 15 min, 4 °C). Supernatants were dried at 95 °C and the cGMP content was measured in duplicate by Radioimmunoassay (RIA). Protein content was determined in pellets used for standardizing the different samples.

### 4.6. Statistical Analysis

All data are expressed as mean ± SEM (*n* = number of animals). Student’s *t*-test was used to compare means of two groups with Gaussian distribution. Differences between dose–response curves were analyzed by one-way or two-way ANOVA for repeated measurements, followed by Bonferroni’s multiple comparison post hoc test. Data of two groups with no Gaussian distribution were analyzed by the Mann–Whitney U test. Probability levels of *p* < 0.05 were considered statistically significant. 

## Abbreviations

ANP	atrial natriuretic peptide
BP	blood pressure
BW	body weight
cGMP	cyclic guanosine monophosphate
DEA-NO	DEA NONOate
GSNO	*S*-nitrosoglutathione
L-NAME	N(G)-Nitro-L-arginine methyl ester
MAP	mean arterial blood pressure
NO	nitric oxide
NO-GC	NO-sensitive guanylyl cyclase (synonym: sGC)
RBF	renal blood flow
SEM	standard error of the mean
SNP	sodium nitroprusside
